# A Call for Dietary Sugar Restriction Policy in India in Light of Oral Health Concerns

**DOI:** 10.7759/cureus.105346

**Published:** 2026-03-16

**Authors:** Saudamini G More, Laresh N Mistry, Amit Patil, Harsh Mishra, Tejal Patil, Ashwin M Jawdekar

**Affiliations:** 1 Public Health Dentistry, Bharati Vidyapeeth (Deemed to be University) Dental College and Hospital, Navi Mumbai, IND; 2 Pediatric and Preventive Dentistry, Bharati Vidyapeeth (Deemed to be University) Dental College and Hospital, Navi Mumbai, IND; 3 Conservative Dentistry and Endodontics, Bharati Vidyapeeth (Deemed to be University) Dental College and Hospital, Navi Mumbai, IND; 4 Orthodontics and Dentofacial Orthopedics, Bharati Vidyapeeth (Deemed to be University) Dental College and Hospital, Navi Mumbai, IND; 5 Oral and Maxillofacial Surgery, Bharati Vidyapeeth (Deemed to be University) Dental College and Hospital, Navi Mumbai, IND

**Keywords:** dental caries, front-of-package (fop) labelling, health policy making, non-communicable diseases (ncds), oral health promotion, primary prevention, regulatory approach, taxation

## Abstract

Rising sugar consumption in India, particularly from sugar-sweetened beverages (SSBs) and ultra-processed foods, has intensified the burden of oral and systemic diseases. Excessive intake of free sugars is strongly linked with dental caries, obesity, type 2 diabetes, and cardiovascular disease, contributing to escalating health and economic costs. Despite global guidelines advocating for reduced free sugar intake, India lacks a comprehensive national strategy to address this shared risk factor for oral and non-communicable diseases (NCDs).

This article integrates international evidence and India-specific data to demonstrate the health and economic impact of sugar consumption and to evaluate the effectiveness of fiscal, regulatory, and educational interventions. Global experiences with excise taxes on SSBs, mandatory front-of-package (FOP) labelling, and restrictions on child-directed marketing have proven successful in reducing sugar intake and prompting industry reformulation. In India, however, policy efforts remain fragmented, with limited taxation and weak regulatory enforcement.

We propose a multisectoral framework tailored to India's epidemiological and socioeconomic context. Recommended measures include a tiered excise tax on high-sugar foods and beverages, mandatory interpretive FOP warning labels, comprehensive bans on marketing to children, school-based sugar control measures, and integration of preventive oral health promotion into primary care. Strengthening surveillance of sugar intake, caries prevalence, and policy outcomes, alongside allocating tax revenues for health promotion and nutrition subsidies, is vital to ensure equity and sustainability.

A comprehensive dietary sugar reduction policy would safeguard children's oral health, reduce future NCD burden, and generate long-term economic and societal benefits for India.

## Introduction and background

India's sugar consumption and the widespread availability of sugar-sweetened products have increased substantially, while dental caries and other sugar-related oral diseases remain highly prevalent and insufficiently addressed within public health strategies [1]. Global guidelines, including those from the World Health Organization (WHO), emphasize the importance of reducing free sugar intake to mitigate both oral and systemic health risks [2].

Recent modelling studies in the Indian context suggest that fiscal and price-related interventions can significantly reduce dental caries prevalence while also generating substantial savings in treatment costs. However, the available evidence on these interventions remains scattered across different disciplines and policy domains [3].

Therefore, a comprehensive policy-focused review is required to amalgamate existing evidence on the impact of sugar-related fiscal and policy interventions on oral health outcomes. This review aims to quantify the effects of such measures on dental caries and evaluate key policy strategies, including taxation, front-of-package (FOP) labelling, and restrictions on marketing. Furthermore, the review seeks to critically assess the relevance and applicability of these interventions within the Indian health system and socioeconomic context. The findings are intended to inform and strengthen policy recommendations for improving oral health outcomes in India.

## Review

Worldwide and Indian disease burden caused by sugar consumption

Excessive consumption of free sugars, and more so from sugar-sweetened beverages (SSBs), is repeatedly linked with excess energy consumption, weight gain, and greater risk of type 2 diabetes (T2D) and cardiometabolic disease. Umbrella reviews and meta-analyses demonstrate strong associations between SSB and T2D and obesity. Emerging evidence implicates high sugar consumption with non-alcoholic fatty liver disease and unfavorable cardiometabolic risk profiles [4-6].

At the population level, SSBs are estimated to add millions of new diabetes and heart disease cases per year in high-consumption regions. In the year 2020, SSBs were estimated to cause 2.2 million new T2D cases and 1.2 million new cardiovascular disease (CVD) cases worldwide [7]. Recent worldwide studies highlight that SSBs are a leading cause of increasing non-communicable disease (NCD) burdens in low- and middle-income countries (LMICs) [8].

India has a rapidly increasing NCD burden. Diabetes prevalence in India is rising rapidly due to T2D, with cases increasing from 32 million in 2000 to 101 million in 2023. The Global Burden of Disease (GBD) 2021 data shows a 62% surge in national incidence from 1990 to 2021. Key drivers include obesity, poor diet, and physical inactivity, with high-burden states like Tamil Nadu and Goa experiencing significant surges. Excess consumption of sugar, in drinks, sweets, and ultra-processed foods, is responsible for this trend, adding to classic risk factors and changing eating habits. Modelling research indicates that population-level decreases in sugar use (for instance, through taxation) in India would prevent huge future incidence of obesity and diabetes [9-11].

In addition to clinical disease, the economic cost of healthcare is high. Excessive consumption of sugar leads to higher demand for curative care, lost productivity, and disability over the long term. Recent policy modelling and cost-of-illness studies highlight the potential for economic benefits from cutting sugar consumption at scale [12,13].

Sugar intake in India

Patterns and per capita availability of sugar intake in India have evolved over the past decades. National and international data (Food and Agriculture Organization/Food and Agriculture Organization Corporate Statistical Database (FAO/FAOSTAT), industry, and academic estimates) reveal per capita consumption of sugar in India at ~17-25 kg/year based on the dataset and time period, around the same or slightly less than the world average but with significant geographical and subpopulation variation. Notably, increased consumption of SSBs and packaged foods has brought into diets high levels of "hidden" free sugars, especially for urban and young populations [14].

Current surveys show that a very high percentage of Indian children and adolescents consume packaged ultra-processed foods and SSBs on a regular basis. In one national survey, more than 90% of children ate packaged ultra-processed foods weekly, and 68% consumed SSBs weekly. This early and frequent exposure leads to cumulative lifetime free sugar intake and risk for caries [15]. There are cultural subtleties like traditional sugars (jaggery, khandsari) and sweets which continue to be significant sources, particularly in certain regional diets, while Western-style SSBs and flavored dairy beverages are gaining market share. These two routes of exposure (traditional sugar foods in addition to packaged SSBs/ultra-processed foods) complicate interventions [16,17].

Influence of sugar intake on oral health

Dental caries is a diet-dependent, biofilm-mediated condition where free sugars are the major substrate for cariogenic bacteria. Sugar fermentation by bacteria in the mouth yields organic acids that demineralize enamel; repeated exposure to sugar results in cumulative demineralization and caries. The Stephan curve illustrates the rapid decrease in plaque pH after sugar consumption and shows how long the dental biofilm remains below the critical pH at which enamel dissolution occurs. Frequent snacking on sugary foods prolongs the period of acid exposure and reduces the opportunity for remineralization of tooth enamel [18,19]. There is a strong evidence base from epidemiologic studies that associates increased free sugar consumption with increased prevalence and severity of caries in all age groups. Systematic reviews and longitudinal studies conclude that decreasing free sugar consumption to less than 5% of total energy intake decreases caries incidence; WHO's guideline in part draws on dental evidence in its recommendations. Benefits appear when the population's intake of sugar is drastically decreased, indicating further gains in energy, though caries cannot be completely avoided at any one threshold due to other causative factors (fluoride exposure, oral hygiene, care access) [18,20,21].

The global prevalence of dental caries was approximately 48%, while that in Asia was 52% approximately [22]. In India, the prevalence of dental caries among children and adults continues to be high with widespread untreated decay and robust socioeconomic gradients. Early childhood caries remains a problem for a large number of young children in spite of prevention knowledge among providers, suggesting failure at the population level to alter dietary and environmental risk factors [23,24].

Oral disease has downstream implications: pain, infection, missed school days, and effects on nutrition, growth, and quality of life. For most Indians, direct out-of-pocket expenditures on dental care create financial hardship, and restricted access to preventive dental care results in the fact that the main effective lever for population-level oral disease reduction lies upstream in the dietary policy [13,25].

Dietary sugar restriction necessity

The WHO advises limiting free sugars during the life course to less than 10% of total energy intake in both adults and children and further lowering it to below 5% for greater benefit in reducing dental caries and energy balance. This advice is based on systematic reviews indicating that reduced free sugar consumption is associated with fewer caries and improved weight outcomes [2]. India's present trends, high SSB and ultra-processed food intake among young people, ongoing high consumption of traditional sugar foods, and low dietary literacy, result in many population subgroups consuming more than these recommended sugar levels. With the lifelong, cumulative nature of dental caries and the simultaneous contribution of sugars to NCDs, a national policy to limit sugar exposure is essential to safeguard children's developing dentition and lower future NCD burden [4,15].

Population-level policy is specially needed since individual behavior change interventions (education, clinical counseling) have low reach and sustainability. Structural interventions that alter the food environment (price, availability, marketing) have wider, equitable coverage and guard at-risk groups who are disproportionately targeted by the aggressive marketing of sugary foods [26,27].

Global policy interventions and evidence

Some nations have introduced SSB excise taxes (Mexico, the UK, South Africa, various European and Latin American nations), FOP labelling, advertising bans to children, and school food standards. Systematic reviews of the rolled-out SSB taxes demonstrate consistent price and sales effects: taxes raise prices and lower buys of taxed products, with declines in sugar bought from beverages tending to equate to declines in population sugar consumption. The UK's Soft Drinks Industry Levy (SDIL) resulted in widespread decreases in sugar bought and prompted reformulation by the industry. Meta-analyses illustrate that multiple real-world SSB taxes decreased sales by different percentage points based on tax design and pass-through [27-29].

A study by Afshin et al. examined the impact of price change on diet in interventional and prospective observational studies largely based in hospital settings. The authors reported a pooled price elasticity of −0.67 (95% CI: −0.31 to −1.04), which means a 7% decrease in consumption for a 10% increase in price [30]. Cabrera Escobar et al. examined the impact of SSB taxes and price changes (for example, in tax simulation modelling) on SSB consumption reporting a combined price elasticity of −1.30 (95% CI: −1.09 to −1.51) [31]. A systematic review of published US simulation studies reported similar price elasticities of −0.79 (95% CI: −0.33 to −1.24) [32].

Growing evidence from LMICs indicates the favorable impacts of taxation and complementary policies on lowering purchasing and possibly preventing obesity and diabetes in modelled scenarios. Most countries have combined taxes with advertising restrictions and warning labels to maximize effectiveness [33-35].

India's policy environment

The existing policy measures related to sugar consumption in India are collated in Table 1.

**Table 1:** Existing policy measures related to sugar consumption in India SSBs: sugar-sweetened beverages; GST: goods and services tax; FOP: front-of-package; FSSAI: Food Safety and Standards Authority of India

Policy measure	Implementing authority	Description	Current status/limitation
Taxation of SSBs under GST	Government of India (GST Council)	Aerated drinks are subject to higher GST and compensation, increasing the retail price of these beverages	Primarily a fiscal measure rather than a dedicated health tax; not explicitly designed to reduce sugar consumption or promote product reformulation
School canteen regulations	Ministry of Health and Family Welfare and state authorities	Guidelines and regulatory actions to limit the availability and sale of unhealthy foods and beverages in school canteens	Implementation varies across states and institutions; largely advisory or voluntary in many settings
Advertising restrictions (in certain jurisdictions)	Government regulatory bodies and state authorities	Efforts to restrict the advertising and promotion of unhealthy food and beverages, particularly targeting children	Limited and inconsistent enforcement; not part of a unified national strategy
FOP labelling	FSSAI	Labelling system to warn consumers about high levels of sugar, salt, and fat in packaged foods	Aimed at improving consumer awareness, but full implementation and effectiveness are lacking
Modelling evidence for health taxes	Academic and policy research institutions	Studies estimate that taxation of SSBs could reduce consumption and prevent future cases of non-communicable diseases	Evidence exists, but translation into comprehensive national policy and evaluation remains limited

As a health education approach for behavior change, "National No Sugar Day" is observed on the first of November in India. National No Sugar Day emerged from the FDI World Dental Federation and the Indian Dental Association's Mumbai Declaration on Sugary Drinks and Healthy Foods (June 2022) [36,37].

Identified gaps and proposed policy roadmap

Priority gaps are the absence of a health-focused excise tax with defined public health aims and earmarking of revenue for health promotion, inadequate coverage of sources of added sugars (excises tend to reach sodas but not other SSBs or sweets), weak regulation of child-directed marketing on all media platforms, FOP labelling that varies and is not high in consumer understanding, and poor inclusion of oral health targets within NCD and nutrition policy agendas [34,38].

Closing the gap demands a multisectoral, evidence-based set of actions that coordinate fiscal, regulatory, educational, and service delivery measures. The next principles are key:

Comprehensiveness

Policies must aim at significant contributors to free sugars (SSBs, packaged food with high levels of sugar, confectionery, flavored dairy) instead of targeting exclusively carbonated drinks. There is evidence that wider coverage brings about greater reductions in exposure to sugar [27,28].

Health Impact and Equity Design

Tax rates should be high enough to change prices (there is evidence that at least 10-20% retail price excise will bring about a significant reduction) and be designed to prevent regressive damage (allocate revenue for the promotion of health and subsidize healthier options). Modelling in India suggests significant population health benefits with well-designed taxes [9,39].

Complementarity Regulation

Integrate fiscal policies with FOP warning labels, strict prohibitions on marketing to children (including online), school canteen rules, and provision of safe drinking water in schools and public areas. These strategies cumulatively support behavior change and limit industry avoidance [27,40].

Integration With Dental Care Services

Consolidate preventive dental health in primary care (fluoride toothpaste promotion, minimal intervention care, and education in early childhood) while employing policy drivers to decrease exposure to sugar as the first line of prevention [26,32].

Monitoring and Research

Establish strong surveillance of sugar intake (total and by source) and prevalence/incidence of caries and monitor policy effects using equity-based indicators. Generation of evidence in India is necessary to tailor policies to local settings [12,41].

From the international evidence and India's epidemiological context, an integrated national dietary sugar limit policy is proposed with the following elements:

Health-Targeted SSB and High-Sugar Foods Excise Tax

An excise tax on all foods and beverages that contain added free sugars above specified levels (including sodas, fruit beverages, sweetened milks, energy drinks, sweetened tea/coffee drinks, and some high-sugar snacks) is to be levied. A "tiered-specific excise" refers to a type of tax imposed by the government where the tax rate is divided into different levels or categories (tiers) depending on the amount of sugar present in a product. Instead of applying the same tax to all products, the tax increases as the sugar content increases. The sugar content is measured as grams of sugar per 100 milliliters (mL) for beverages or per 100 grams (g) for solid food products. The purpose of implementing such a tiered tax system is to encourage reformulation. Reformulation states that food and beverage manufacturers are motivated to modify their product recipes by reducing the amount of sugar. Additionally, the policy should aim for at least a 20% increase in the average retail price of high-sugar products. The expected outcome of this price increase is to bring about behavioral change among consumers. Evidence favors tiered sugar taxes for encouraging reformulation, as in the UK [26,29]. Set aside a large share of revenues for primary prevention (school nutrition program, community oral health promotion, safe drinking water in schools) and for healthy food subsidies (fruit, dairy without added sugar). Earmarking revenue lessens political resistance and weakens regressive consequences [39].

Mandatory FOP Warning Labels and Strong Advertising Controls

Implement mandatory interpretive FOP warning labels (black stop-sign or octagon labels) for products exceeding thresholds for free sugar, salt, or saturated fat. Evidence suggests FOP warnings improve consumer understanding and can reduce purchases [25]. Ban child-targeted marketing of high-sugar foods through TV, online media, point-of-sale promotions, and influencer marketing; mandate time/age constraints and pre-approval of child-directed marketing content. Marketing bans should extend to digital media where youth viewing is extensive [26].

School-Based Measures

Mandate sugar-free school cafeterias, water-only rules, nutrition education in the curriculum, and regular monitoring. Schools are an important place for early prevention and breaking routine consumption habits [23,26].

Integration of Primary Care and Community

Integrate oral health promotion within primary healthcare (antenatal care/child health visits), with focus on counselling on sugar reduction, brushing with fluoridated toothpaste, and early childhood caries prevention. Capacity building of frontline health workers (Accredited Social Health Activists (ASHAs), Anganwadi workers) for the provision of brief behavioral interventions is cost-effective and scalable [23,38].

Stakeholder Involvement and Resisting Industry Interference

Construct cross-sectoral alliances (health, finance, education, consumer affairs) and safeguard policymaking from commercial interests with conflict-of-interest protection. Public outreach campaigns should present health justifications and tax revenue expenditure for child health [27,38].

Implementation considerations and potential challenges

Industry Resistance and Reformulation

Industry players regularly campaign against taxes and controls. Nevertheless, evidence indicates that well-designed taxes trigger reformulation and decrease sugar quantities sold, which is a policy triumph even as revenue declines in the long run. A tiered tax on sugar content induces product reformulation to decrease sugar levels [28,29].

Equity and Affordability Issues

Excise taxes may be regressive in form but progressive in effect if revenues are spent on health programs aimed at low-income populations and if taxes cut disease burden in the most disadvantaged. Subsidies on healthy foods and on school programs can address fairness issues [39].

Scope and Definitions

Clear regulatory definitions of "free sugars" and the food products subject to taxation are important to avoid loopholes in policy implementation. Policies should cover a wide range of foods that contain added sugars, such as confectionery, flavored dairy products, and certain baked goods. Including multiple sources of added sugars helps prevent consumers from simply switching to other sweet foods that are not covered by the regulation [38].

Enforcement and Capacity in Tax Administration

India has indirect tax experience (goods and services tax (GST)) and can apply existing tax administration but may require the development of specific health tax enforcement and tracking of sugar levels. Finance and health ministry cooperation is critical [35].

Expected impact and cost-effectiveness

Synthesizing international evaluations and India-specific modelling suggests that a comprehensive policy package, taxation with earmarking, FOP labelling, marketing restrictions, and school policies, would reduce population sugar intake, lower dental caries incidence (particularly in children), reduce SSB-attributable obesity and diabetes, and be cost-saving or cost-effective from a societal perspective due to averted healthcare costs and productivity gains. Modelling analyses suggest significant reductions in obesity and diabetes incidence with a 20% tax on SSBs; real-world data demonstrate quantifiable declines in sales following implementation [28,29,39].

Limitations

This review has certain limitations. As a narrative policy-focused review, it did not follow a formal systematic review protocol, and some relevant studies may not have been captured. Additionally, much of the evidence on sugar reduction policies such as taxation and FOP labelling comes from international contexts, which may limit direct applicability to India. Furthermore, India-specific evidence evaluating the impact of these policies on oral health outcomes remains limited, and some interpretations rely on modelling studies and broader public health literature. The review also primarily focuses on national-level policy measures, which may not fully reflect regional- or state-level initiatives.

## Conclusions

Dental health and systemic NCDs both have in common a modifiable shared risk factor: exposure to free sugar. In India, shifting eating patterns and widespread SSB and ultra-processed food intake among children and adolescents require bold, multisectoral policy interventions. International evidence shows that fiscal and regulatory interventions can lower sugar purchases and drive industry reformulation. India must implement an evidence-based dietary sugar limitation policy, pairing health-focused excise taxation, required interpretive FOP labelling, child marketing restrictions, school nutrition guidelines, and primary care incorporation of oral health prevention, with revenues spent on health promotion and equity initiatives. Such a package would safeguard children's oral health, add to NCD avoidance, and bring huge long-term well-being and economic benefits.
